# Prevention of mother-to-child transmission of HIV: a cross-sectional study in Malawi

**DOI:** 10.2471/BLT.17.203265

**Published:** 2018-02-28

**Authors:** M van Lettow, M Landes, JJ van Oosterhout, E Schouten, H Phiri, E Nkhoma, T Kalua, S Gupta, N Wadonda, A Jahn, B Tippett-Barr

**Affiliations:** a Dignitas International, PO Box 1071, Zomba, Malawi.; b Department of Family and Community Medicine, University of Toronto, Toronto, Canada.; c Management Sciences for Health, Lilongwe, Malawi.; d Department of HIV and AIDS, Ministry of Health, Lilongwe, Malawi.; e United States Centers for Disease Control and Prevention – Lilongwe, Lilongwe, Malawi.; f United States Centers for Disease Control and Prevention – Zimbabwe, Harare, Zimbabwe.

## Abstract

**Objective:**

To estimate the use and outcomes of the Malawian programme for the prevention of mother-to-child transmission (MTCT) of human immunodeficiency virus (HIV).

**Methods:**

In a cross-sectional analysis of 33 744 mother–infant pairs, we estimated the weighted proportions of mothers who had received antenatal HIV testing and/or maternal antiretroviral therapy and infants who had received nevirapine prophylaxis and/or HIV testing. We calculated the ratios of MTCT at 4–26 weeks postpartum for subgroups that had missed none or at least one of these four steps.

**Findings:**

The estimated uptake of antenatal testing was 97.8%; while maternal antiretroviral therapy was 96.3%; infant prophylaxis was 92.3%; and infant HIV testing was 53.2%. Estimated ratios of MTCT were 4.7% overall and 7.7% for the pairs that had missed maternal antiretroviral therapy, 10.7% for missing both maternal antiretroviral therapy and infant prophylaxis and 11.4% for missing maternal antiretroviral therapy, infant prophylaxis and infant testing. Women younger than 19 years were more likely to have missed HIV testing (adjusted odds ratio, aOR: 4.9; 95% confidence interval, CI: 2.3–10.6) and infant prophylaxis (aOR: 6.9; 95% CI: 1.2–38.9) than older women. Women who had never started maternal antiretroviral therapy were more likely to have missed infant prophylaxis (aOR: 15.4; 95% CI: 7.2–32.9) and infant testing (aOR: 13.7; 95% CI: 4.2–83.3) than women who had.

**Conclusion:**

Most women used the Malawian programme for the prevention of MTCT. The risk of MTCT increased if any of the main steps in the programme were missed.

## Résumé

**Objectif:**

Évaluer l'utilisation et les résultats du programme malawite pour la prévention de la transmission mère-enfant (TME) du virus de l'immunodéficience humaine (VIH).

**Méthodes:**

Dans le cadre d'une analyse transversale de 33 744 paires mère-nourrisson, nous avons estimé la part pondérée des mères qui avaient bénéficié d'un dépistage prénatal du VIH et/ou d'un traitement antirétroviral maternel et la part pondérée des nourrissons qui avaient bénéficié d'un traitement prophylactique par la névirapine et/ou d'un dépistage du VIH. Nous avons calculé les rapports de TME à 4-26 semaines post-partum pour des sous-groupes qui n'avaient manqué aucune de ces quatre étapes ou qui en avaient manqué au moins une.

**Résultats:**

Le recours à un dépistage prénatal a été estimé à 97,8%, le recours à un traitement antirétroviral maternel à 96,3%, le recours à un traitement prophylactique pour nourrisson à 92,3% et le recours à un dépistage du VIH chez le nourrisson à 53,2%. Les rapports de TME ont été estimés à 4,7% en tout et à 7,7% dans le cas des paires qui avaient manqué le traitement antirétroviral maternel, à 10,7% dans le cas des paires qui avaient manqué le traitement antirétroviral maternel et le traitement prophylactique pour nourrisson, et à 11,4% dans le cas des paires qui avaient manqué le traitement antirétroviral maternel, le traitement prophylactique pour nourrisson et le dépistage du VIH chez le nourrisson. Les femmes âgées de moins de 19 ans étaient davantage susceptibles d'avoir manqué le dépistage du VIH (rapport des cotes ajusté, RCa: 4,9; intervalle de confiance, IC, à 95%: 2,3-10,6) et le traitement prophylactique pour nourrisson (RCa: 6,9; IC à 95%: 1,2-38,9) que les femmes plus âgées. Les femmes qui n'avaient jamais commencé de traitement antirétroviral maternel étaient davantage susceptibles d’avoir manqué le traitement prophylactique pour nourrisson (RCa: 15,4; IC à 95%: 7,2-32,9) et le dépistage du VIH chez le nourrisson (RCa: 13,7; IC à 95%: 4,2-83,3) que les autres femmes.

**Conclusion:**

Une majorité de femmes a eu recours au programme malawite pour la prévention de la TME. Le risque de TME augmentait lorsque l'une des principales étapes du programme n'était pas suivie.****

## Resumen

**Objetivo:**

Estimar el uso y los resultados del programa malawi para la prevención de la transmisión de madre a hijo (MTCT) del virus de inmunodeficiencia humana (VIH)

**Métodos:**

En un análisis transversal a 33 744 pares de madres y recién nacidos, estimamos las proporciones ponderadas de madres que han realizado el test de VIH prenatal y/o terapia antirretroviral maternal y los recién nacidos que han recibido profilaxis con nevirapina y/o el test de VIH. Calculamos los coeficientes de MTCT en las semanas 4 a 26 de postparto para subgrupos que no habían faltado a ninguno o al menos a uno de esos cuatro pasos.

**Resultados:**

La aceptación estimada del test prenatal fue del 97,8% mientras que la de la terapia antirretroviral maternal fue del 96,3%; la de profilaxis en recién nacidos fue del 92,3% y la del test de VIH en recién nacidos fue del 53,2%. Los coeficientes estimados de MTCT fueron del 4,7% en general y del 7,7% para los pares que habían faltado a terapia antirretroviral maternal, del 10,7% para los que faltaron a ambas, la terapia antirretroviral maternal y la profilaxis en recién nacidos; y del 11,4% para los que faltaron a terapia antirretroviral maternal, a la profilaxis en recién nacidos y al test de recién nacidos. Las mujeres menores de 19 años presentaban mayores probabilidades de haber faltado al test de VIH. (Coeficiente de posibilidades ajustado, CPa: 4,9; intervalo de confianza, IC del 95%: 2,3-10,6) profilaxis en recién nacidos (CPa: 6,9; IC del 95%: 1,2-38,9) que en mujeres mayores. Las mujeres que nunca empezaron la terapia antirretroviral maternal presentaban mayores probabilidades de haber faltado a la profilaxis de recién nacidos (CPa: 15,4; IC del 95%: 7,2-32,9) y al test de recién nacidos (CPa: 13,7; IC del 95%: 4,2-83,3) que las mujeres que habían empezado.

**Conclusión:**

La mayoría de las mujeres usaron el programa malawi para la prevención de MTCT. El riesgo de MTCT aumenta si falta alguno de los pasos principales del programa.

## ملخص

**الغرض:**

تقييم استخدام البرنامج الملاوي ونتائجه للوقاية من انتقال فيروس عوز المناعة البشري (HIV) من الأم إلى الطفل (MTCT).

**الطريقة:**

أجرينا تحليلاً متعدد القطاعات لحوالي 33744 زوجًا من الأمهات والرضع، وقدرنا من خلاله النسب المرجحة للأمهات اللاتي خضعن لاختبار الكشف عن الإصابة بفيروس عوز المناعة البشري في فترة الحمل و/أو اللاتي تلقين العلاج باستخدام مضادات الفيروسات القهقرية، وكذلك الأطفال الذين تلقوا علاج الوقاية بالنيفيرابين و/أو خضعوا لاختبار الكشف عن هذا الفيروس. قمنا بحساب نسب انتقال العدوى من الأم إلى الطفل في الفترة فيما بين 4 إلى 26 أسبوعًا بعد الولادة للفئات الفرعية التي لم تفوت الخطوات الأربعة أو فوتت خطوة واحدة على الأقل.

**النتائج:**

كانت نسبة الاستيعاب المقدر للاختبار في فترة الحمل 97.8‏‏%؛ في حين كانت نسبة العلاج باستخدام مضادات الفيروسات القهقرية 96.3%؛ ونسبة علاج وقاية الأطفال 92.3‏‏%؛ ونسبة اختبار فيروس عوز المناعة البشري للأطفال 53.2‏‏%. كانت النسب المقدرة لانتقال الفيروس من الأم إلى الطفل 4.7‏‏% إجماليًا و‏7.7% للأزواج الذين فوتوا العلاج باستخدام مضادات الفيروسات القهقرية، و‏‏10.7‏% للاتي فوتن كلًا من العلاج باستخدام مضادات الفيروسات القهقرية وعلاج وقاية الأطفال، و‏11.4% لمن فوتن العلاج باستخدام مضادات الفيروسات القهقرية وعلاج وقاية الأطفال واختبار الأطفال. تكون النساء الأصغر من 19 عامًا أكثر عرضة لتفويت اختبار فيروس عوز المناعة البشري (نسب الاحتمالية المعدلة aOR‏: 4.9؛ بنسبة أرجحية مقدارها ‏‏95‏%: ‏2.3–10.6) وعلاج وقاية الأطفال (aOR‏: 6.9؛ ‏95‏% كمقدار لنسبة الأرجحية: 1.2‏–38.9) من النساء الأكبر سنًا. تكون النساء اللاتي لم يبدأن العلاج باستخدام مضادات الفيروسات القهقرية أكثر عرضة لتفويت علاج وقاية الأطفال (aOR‏: 15.4؛ ‏95‏‏% كمقدار لنسبة الأرجحية: 7.2‏-32.9) واختبار الأطفال (‏aOR: 13.7؛ ‏95‏% كمقدار لنسبة الأرجحية: 4.2–83.3) من النساء اللاتي بدأن فيه.

**الاستنتاج:**

تستخدم معظم النساء البرنامج الملاوي للوقاية من انتقال الفيروس من الأم إلى الابن. تزداد مخاطر انتقال الفيروس من الأم إلى الطفل إذا تم تفويت أية خطوة من خطوات البرنامج.

## 摘要

**目的:**

评估马拉维共和国人类免疫缺陷病毒 （HIV，又称艾滋病毒）母婴传播 (MTCT) 预防计划的使用和结果。

**方法:**

在 33 744 组母婴的横断面分析中，我们估算了接受过产前艾滋病毒检测和/或孕产妇抗逆转录病毒治疗的母亲以及接受过奈韦拉平预防和/或艾滋病毒检测的婴儿的权重比例。我们计算了在产后 4 至 26 周四个步骤无一遗漏或至少遗漏一个的亚群体的母婴传播 (MTCT) 的比例。

**结果:**

产前检测的估计值为 97.8%；孕产妇抗逆转录病毒治疗为 96.3%；婴儿预防治疗为 92.3%；婴儿艾滋病毒检测为 53.2%。母婴传播 (MTCT) 的总体估计比例为 4.7%，遗漏孕产妇抗逆转录病毒治疗的母婴组为 7.7%，遗漏孕产妇抗逆转录病毒治疗和婴儿预防治疗的为 10.7%，遗漏孕产妇抗逆转录病毒治疗、婴儿预防治疗和婴儿测试的为 11.4%。相较于年长女性，19 岁以下的女性更容易遗漏艾滋病毒检测（调整后比值，aOR：4.9；95% 置信区间，CI：2.3–10.6） 和婴儿预防治疗 （aOR：6.9；95% CI：1.2–38.9）。从未接受过抗逆转录病毒治疗的女性比接受过抗逆转录病毒治疗的女性更有可能遗漏婴儿预防治疗（aOR：15.4；95% CI：7.2-32.9） 和婴儿检测 （aOR：13.7；95% CI：4.2–83.3）。

**结论:**

大多数女性参与了预防人类免疫缺陷病毒母婴传播 (MTCT) 的马拉维预防计划。如果遗漏了该预防计划的任何主要步骤，母婴传播 (MTCT) 的风险就会增加。****

## Резюме

**Цель:**

Оценить использование и результаты малавийской программы профилактики передачи вируса иммунодефицита человека (ВИЧ) от матери ребенку (ПМР).

**Методы:**

В ходе кросс-секционного анализа 33 744 пар мать-младенец авторы рассчитали взвешенные пропорции матерей, которые прошли антенатальное тестирование на ВИЧ и (или) получали антиретровирусную терапию, и младенцев, которые получали невирапин в качестве профилактики и (или) прошли тестирование на ВИЧ. Авторы рассчитали долю ПМР на 4–26-й неделе после родов для подгрупп, которые не пропустили ни одного этапа программы или у которых отсутствовал как минимум один из этих четырех этапов.

**Результаты:**

Расчетный показатель использования антенатального тестирования составил 97,8 %, в то время как показатель использования антиретровирусной терапии у матерей составил 96,3 %, показатель детской профилактики — 92,3 % и показатель тестирования на ВИЧ среди новорожденных — 53,2 %. Расчетные показатели ПМР составили 4,7 % в целом и 7,7 % для пар, в которых мать не получала антиретровирусную терапию, 10,7 % для пар, в которых не проводились ни антиретровирусная терапия матери, ни профилактика младенца, и 11,4 % для пар, в которых не проводились антиретровирусная терапия матери, профилактика и тестирование для младенца. У женщин моложе 19 лет чаще отсутствовали тестирование на ВИЧ (скорректированное отношение шансов, сОШ: 4,9; 95%-ный доверительный интервал, ДИ: 2,3–10,6) и профилактика для младенца (сОШ: 6,9; 95%-ный ДИ: 1,2–38,9) по сравнению с женщинами старше 19 лет. У младенцев, чьи матери никогда не получали антиретровирусную терапию, чаще всего отсутствовала профилактика ПМР (сОШ: 15,4; 95%-ный ДИ: 7,2–32,9) и тестирование на ВИЧ (сОШ: 13,7; 95%-ный ДИ: 4,2–83,3) по сравнению с младенцами, чьи матери получали такую терапию.

**Вывод:**

Большинство женщин использовали малавийскую программу профилактики ПМР. Риск ПМР повышался, если они пропускали какой-либо из основных этапов программы.

## Introduction

Recent global efforts in the fight against human immunodeficiency virus (HIV) have been focused on the virtual elimination of paediatric HIV infection^1^ and improvements in the so-called cascade of care for the prevention of mother-to-child transmission of HIV (PMTCT). This cascade includes antenatal HIV testing, uptake of maternal antiretroviral therapy (ART), infant antiretroviral prophylaxis and early infant HIV testing.^2^

In 2011, Malawi adapted the relevant World Health Organization (WHO) guidelines to design a national, public-health-oriented strategy for PMTCT.^3^^,^^4^ In this strategy, called Option B+, all pregnant and breastfeeding women found infected with HIV are offered lifelong ART, regardless of their CD4+ T-lymphocyte counts or WHO clinical stage. The strategy’s development was supported by emerging evidence that universal ART provision in resource-constrained settings could markedly reduce HIV transmission.^5^^–^^7^ The strategy was designed to remove institutional barriers to care, provide maternal health benefits, reduce maternal mortality and increase ART coverage for future pregnancies in high-fertility settings.^4^ During the strategy’s early implementation, ART initiations among pregnant women increased sixfold and the proportions of women who, having initiated ART while pregnant, were still receiving HIV care 12 and 24 months later were 72% and 68%, respectively.^8^^,^^9^

Although a few studies in large Malawian health facilities have indicated that many mother–infant pairs miss one or more of the four main steps in the cascade of care provided, for PMTCT, by the national HIV programme, the results of such studies cannot be considered nationally representative.^10^^,^^11^ The summary reports produced by Malawi’s national HIV programme use data that are aggregated at health-facility level and do not provide insight into individual risk factors associated with the use of the cascade of care for PMTCT. In 2014, the Malawi Ministry of Health began a national evaluation of Malawi’s PMTCT programme. Here we present some of the methods, results and conclusions of that evaluation, which aimed to determine the effectiveness of the Option B+ strategy in a nationally-representative sample of mother–infant pairs enrolled at 4–26 weeks postpartum.

## Methods

### Study setting

Implementation of the Malawi integrated PMTCT/ART guidelines began in July 2011. In theory, this gave all pregnant and breastfeeding women access to HIV testing, HIV counselling and ART. At the time of HIV status ascertainment, each HIV-infected pregnant woman should be given enough nevirapine to provide her baby with six weeks of prophylaxis from birth. She should also be asked to bring her child, for HIV testing, to a clinic for the care of children younger than five years, known as an under-5 clinic in Malawi, as soon as the course of prophylaxis is complete at an age of six weeks.^4^

### Study design and sampling

Our aim was to draw a representative sample for national estimates of the ratios of mother-to-child transmission of HIV (MTCT) in Malawi. The sampling frame included all 579 health facilities that provided PMTCT services in Malawi in 2012–2013. We estimated that we would need to enrol at least 3376 HIV-exposed infants to determine the ratio of MTCT at 24 months postpartum reliably. Probability-proportional-to-size selection was used, without replacement, to select the 54 study facilities: 14 rural and nine urban facilities in the North or Central regions and 22 rural and nine urban in the South region. We subjected data obtained at all 54 study facilities to a cross-sectional analysis.

### Data collection and laboratory procedures

Between October 2014 and May 2016, women attending under-5 clinics at each of the study facilities were screened for study eligibility. To be enrolled, a woman had to be a mother of or a legal caregiver for an infant aged 4–26 weeks and be willing and able to give informed consent. Information about age, parity, uptake of antenatal care, HIV testing and whether the woman’s HIV status had been ascertained during or before the index pregnancy, if ever, was collected in standardized interviews. Whenever possible, interviewers checked the mothers’ health booklets to check the accuracy of the mothers’ responses. Women who had only discovered that they were HIV-infected through study screening were not asked about their uptake of maternal ART, infant nevirapine prophylaxis or infant HIV testing. After being interviewed, each enrolled woman was tested, within the study facility, for HIV. Maternal HIV testing, which was based on an initial rapid Determine HIV-1/2 test (Alere Medical, Tokyo, Japan) and confirmation with Unigold HIV-1/2 (Trinity Biotech, Bray, Ireland), followed national guidelines.^12^ The Joint Clinical Research Centre in Kampala, Uganda, performed qualitative tests for HIV-1 deoxyribonucleic acid (DNA), based on COBAS AmpliPrep and version 2.0 of the COBAS TaqMan assay (Roche Diagnostics, Indianapolis, United States of America), on batched dried spots of blood from all identified HIV-exposed infants.

### Statistical analyses

We focused on five main steps in the PMTCT cascade of care: attendance at an antenatal clinic – known as step 0; ascertainment of HIV status during antenatal care (step 1); uptake of maternal ART (step 2); use of infant nevirapine prophylaxis (step 3); and HIV testing, before the study, of HIV-exposed infants when more than eight weeks old (step 4). The denominators used to calculate weighted proportions for step 1, steps 2 and 3 and step 4 were, respectively, the total number of mother–infant pairs included in the cohort, the total number of known HIV-infected mothers and the total number of known HIV-infected mothers with infants that were more than eight weeks old, i.e. with infants that should have been tested for HIV. Mothers who claimed to be HIV-negative and were subsequently found negative in the rapid test were categorized as confirmed HIV-uninfected. Similarly, mothers who claimed to be HIV-positive and were subsequently found positive in the rapid test were categorized confirmed HIV-infected. The HIV status of the other mothers was categorized either as missed HIV diagnosis, if the mother claimed to be HIV-negative or not know her HIV status, but was subsequently found positive in the rapid test, or as inconclusive, if the rapid test results were inconclusive. We recorded ratios of MTCT at 4–26 weeks postpartum as the percentage of infants tested for HIV-1 DNA that were found positive. We calculated an overall MTCT ratio and also separate ratios for the mother–infant pairs who had missed none or one or more PMTCT cascade steps or who were categorized as missed HIV diagnosis.

We report unweighted numbers and weighted categorical proportions with 95% confidence intervals (CI). Missing data were treated as additional categories. We used *χ^2^* tests to compare weighted MTCT ratios. We used a weighted multivariable binary logistic regression to identify factors associated with missing steps 1, 2, 3 and/or 4 of the cascade of care or a missed HIV diagnosis. In each model, weighted odds ratios with 95% CI were adjusted for region and maternal age, parity and uptake of antenatal care, at the study site or a different site, to give adjusted odds ratios (aOR). In the models for missed maternal ART uptake, missed nevirapine prophylaxis and missed infant HIV testing, we also adjusted for ascertained maternal HIV status. We also adjusted for maternal ART status and timing in the model for missed uptake of nevirapine prophylaxis and for uptake of nevirapine prophylaxis in the model for missed infant HIV testing. All analyses were conducted using SPSS Statistics 23 (IBM, Chicago, USA), and adjusted for the complex design of the whole national evaluation of Malawi’s PMTCT programme. Each observation was weighted according to sampling interval and the probabilities of districts, clusters and subjects being selected.^13^

### Ethics

Ethical approval was provided by Malawi’s National Health Sciences Research Committee (#1262), the United States Centers for Disease Control and Prevention (#2014–054–7) and the University of Toronto (#30448). All mothers or caregivers provided written informed consent.

## Results

Although 34 637 mothers or caregivers were interviewed and tested for HIV infection, 657 (1.9%) had to be excluded, because of non-eligibility or incomplete data. All 236 (0.7%) caregiver–infant pairs had to be excluded because the caregivers gave inconsistent answers to the questions on PMTCT services. The remaining 33 744 mothers, who attended care with infants aged 4–26 weeks old, were included in our analysis.

### Maternal characteristics

The characteristics of the enrolled mothers and their uptake of each step in the PMTCT programme are summarized in Table 1. All percentages and aOR reported below are weighted values. Mothers’ ages ranged from 12 to 53 years; 17 931 (53.8%) were under 25 years of age and 6247 (20.5%) were adolescents aged 12–19 years. Parity ranged from 1 to 14 and 10 943 (30.5%) of the mothers were primiparous.

**Table 1:** Characteristics of mother–infant pairs visiting clinics for children younger than five years, Malawi, October 2014–May 2016

Characteristic	Unweighted no.	Weighted percentage^a^ (95% CI)
**Location**		
Recorded^b^	33 744	N/A
Rural facility in Central or North regions	8 991	23.2 (15.0–34.2)
Urban facility in Central or North regions	13 748	20.5 (12.1–32.4)
Rural facility in South region	6 106	50.9 (37.6–64.1)
Urban facility in South region	4 899	5.4 (3.6–8.1)
**Mother’s age in years**		
Recorded^b^	33 744	N/A
≤ 19	6 427	20.5 (19.1–22.1)
20–24	11 504	33.3 (32.5–34.1)
25–29	7 423	19.7 (15.8–21.1)
≥ 30	8 315	26.3 (25.2–27.4)
Unknown^c^	75	0.2 (0.1–0.3)
**Age of last born child in weeks**		
Recorded^b^	33 744	N/A
4–12	22 170	63.1 (60.2–35.9)
13–26	11 574	36.9 (34.1–39.8)
**Type of last birth**		
Recorded^b^	33 744	
Singleton	33 579	99.1 (98.7–99.4)
Multiple	165	0.9 (0.6–1.3)
**Parity**		
Recorded^b^	33 744	N/A
1	10 943	30.5 (29.0–32.0)
2–3	14 179	39.6 (38.6–40.7)
4–6	7 810	27.1 (25.5–28.7)
7–9	717	2.7 (2.1–3.4)
≥ 10	31	0.1 (0.1–0.1)
Unknown^c^	64	0.2 (0.1–0.3)
**ANC attendance**		
Recorded^b^	33 744	N/A
Did attend ANC during last pregnancy	33 681	99.8 (99.7–99.9)
Missed ANC uptake	63	0.2 (0.1–0.3)
**Site of ANC uptake**		
Recorded^b^	33 744	N/A
Same site as enrolment site	26 225	76.3 (70.8–81.0)
Other site	7 449	23.5 (18.7–29.0)
Unknown^c^	70	0.2 (1.2–0.3)
**HIV status ascertained at ANC**		
Recorded^b^	33 744	N/A
Found HIV-negative during index pregnancy	30 043	88.5 (86.8–90.0)
Known to be HIV-positive before index pregnancy	1 637	6.4 (5.6–7.3)
Found HIV-positive during index pregnancy	1 596	5.1 (4.3–6.2)
Missed having HIV status ascertained at ANC or unwilling to reveal result	468	2.2 (1.1–4.4)
**Maternal HIV status 4–26 weeks postpartum**		
Recorded^b^	33 744	N/A
Confirmed HIV-uninfected	30 057	87.5 (85.5–89.0)
Confirmed HIV-infected	3 233	11.3 (9.8–12.9)
Newly identified HIV-infected	286	0.9 (0.6–1.2)
Had inconclusive results	168	0.4 (0.5–0.5)
**ART status and timing of ART initiation among confirmed HIV-infected**		
Recorded^b^	3 233	N/A
On ART since before index pregnancy	1 572	54.0 (49.9–57.9)
On ART, having started during index pregnancy	1 475	40.7 (35.6–46.0)
On ART, having started postpartum	49	1.6 (0.9–2.9)
Off ART, having started and stopped	34	0.6 (0.3–1.1)
Off ART and never started	50	1.2 (0.6–2.4)
Unknown^c^	53	1.9 (0.7–4.9)
**Infant nevirapine prophylaxis given to known HIV-exposed infants**		
Recorded^b^	3 233	N/A
From birth to an age of 6 weeks	2 676	75.9 (66.3–83.5)
For less than 6 weeks	323	16.4 (9.5–26.9)
Missed indicated nevirapine prophylaxis uptake or unwilling to reveal	234	7.7 (6.1–9.6)
**Uptake of early infant diagnosis among HIV-exposed infants over 8 weeks of age**		
Recorded^b^	1 465	N/A
Tested at an age of at least 6 weeks	790	53.2 (46.3–60.0)
Not tested	675	46.8 (40.0–53.7)
**HIV status of HIV-exposed infants aged 4–26 weeks**		
Recorded^b^	3 519	N/A
HIV-uninfected	3 345	95.3 (93.6–96.6)
HIV-infected	174	4.7 (3.4–6.3)

ANC: antenatal clinic; ART: antiretroviral therapy; CI: confidence interval; HIV: human immunodeficiency virus; N/A: not applicable.

^a^ Weighted according to sampling interval and the probabilities of districts, clusters and subjects being selected.

^b^ Number used as unweighted denominator.

^c^ Unknown because the relevant question was not answered, the recorded answer could not be read or multiple answers were recorded when only one was allowed.

During the index pregnancy, 33 681 (99.8%) of the mothers attended an antenatal clinic (step 0), 33 276 (97.8%) had their HIV status ascertained at such a clinic (step 1) and 26 225 (76.3%) reported that they had attended an antenatal clinic at the site where they were enrolled.

Of the women who reported that they had had their HIV status ascertained at an antenatal clinic, 1637 (6.4%) and 1596 (5.1%) reported that they had been found HIV-positive before and during the index pregnancy, respectively.

HIV testing at the time of our study identified 3519 (12.1%) mothers with HIV infection, including 3233 (11.3%) confirmed HIV infections and 286 (0.9%) missed HIV diagnoses. The latter represented by 244 mothers who had claimed they were HIV-negative and 42 who had claimed not to know their HIV status.

Of the 3233 confirmed HIV-infected women, 3096 (96.3%) were on ART (step 2); 1572 (54.0%) had started ART before the index pregnancy, 1475 (40.7%) during the index pregnancy and 49 (1.6%) postpartum. Thirty-four (0.6%) of the confirmed HIV-infected women had stopped ART and 50 (1.2%) had not started ART. For 53 (1.9%) women the ART status was unknown.

Of the confirmed HIV-infected mothers, 2676 (75.9%) reported giving their infant nevirapine prophylaxis from birth to an age of six weeks (step 3) and 323 (16.4%) had given such prophylaxis for less than six weeks. Although, when they were interviewed, 139 of the 323 had infants that were under six weeks of age. Of the 1465 identified HIV-exposed infants that were older than eight weeks when their mothers were enrolled, 790 (53.2%) had been tested for HIV-1 DNA before the study screening (step 4).

The overall ratio of MTCT at 4–26 weeks, among 3519 HIV-exposed mother–infant pairs, was 4.7%.

### Characteristics associated with missed steps

Overall, 468 (2.2%) mothers claimed that they had not had their HIV status ascertained during antenatal care. In multivariable analysis, adolescent mothers (aOR: 4.9; 95% CI: 2.3–10.6) and those aged 20–24 years (aOR: 2.0; 95% CI: 1.4–2.8) were more likely to have missed this step than older mothers and mothers who had had two or three (aOR: 2.1; 95% CI: 1.2–3.7) or at least four (aOR: 2.5; 95% CI: 1.3–4.6) previous deliveries were more likely to have missed this step than primiparous mothers (Table 2).

**Table 2:** Factors associated with missing antenatal testing for HIV infection and with first identification of such infection 4–26 weeks postpartum, Malawi, October 2014–May 2016

Characteristic	Unweighted denominator	Missed antenatal HIV testing			Newly identified HIV infections^a^	
		Unweighted, no. (%)	Weighted aOR^b^ (95% CI)		Unweighted, no. (%)	Weighted aOR^b^ (95% CI)
**Location (*n* = 33 744)**			NIIM^c^			
Rural facility in Central or North regions	8 991	73 (0.8)			54 (0.6)	0.8 (0.4–1.6)
Urban facility in Central or North regions	13 748	80 (0.6)			96 (0.7)	0.5 (0.2–1.1)
Rural facility in South region	6 106	278 (4.6)			52 (0.9)	1.0 (0.4–2.2)
Urban facility in South region	4 899	37 (0.8)			42 (0.9)	Reference
**Mother’s age in years (*n* = 33 669)**						
≤ 19	6 427	119 (1.9)	4.9 (2.3–10.6)		24 (0.4)	0.6 (0.3–1.3)
20–24	11 504	232 (2.0)	2.0 (1.4–2.8)		93 (0.8)	1.1 (0.6–2.2)
25–29	7 423	105 (1.4)	1.3 (0.8–1.5)		66 (0.9)	1.1 (0.6–2.1)
≥ 30	8 315	11 (0.1)	Reference		61 (0.7)	Reference
**Parity (*n* = 33 680)**						
1	10 943	139 (1.3)	Reference		42 (0.4)	1.0 (0.5–2.0)
2–3	14 179	186 (1.3)	2.1 (1.2–3.7)		148 (1.0)	2.0 (1.2–3.6)
≥ 4	8 558	143 (1.7)	2.5 (1.3–4.6)		54 (0.6)	Reference
**ANC attendance (*n* = 33 744)**			NIIM^d^			NIIM^d^
Yes	33 681	425 (1.3)			244 (0.7)	
No	63	43 (68.3)			0 (0.0)	
**Location of ANC (*n* = 33 674)**						
At study site	26 225	349 (1.3)	Reference		163 (0.6)	Reference
At different site	7 449	77 (1.0)	0.7 (0.3–1.9)		81 (1.1)	2.2 (1.5–3.1)

ANC: antenatal clinic; aOR: adjusted odds ratio; CI: confidence interval; HIV: human immunodeficiency virus; NIIM: not included in model.

^a^ Mother missed HIV diagnosis, she claimed to be HIV-negative or not to know her HIV status, but was found infected in the study.

^b^ Weighted according to sampling interval and the probabilities of districts, clusters and subjects being selected and adjusted for all other variables in the model.

^c^ Excluded from model because of skewed data caused by three rural health facilities in South region having no test kits available when women would have been seeking antenatal care.

^d^ Too few events to be included in the model.

Of the 3519 HIV-infected women identified at screening, 286 (7.1%) had missed being diagnosed earlier. Such missed diagnosis was associated with having attended an antenatal clinic somewhere other than the study site (aOR: 2.2; 95% CI: 1.5–3.1) and with parity, with an aOR of 2.0 (95% CI: 1.2–3.6) for a parity of 2–3 compared with one of at least 4 (Table 2).

Fifty (1.2%) of the 3233 confirmed HIV-infected women had not started ART (Table 3). Not having started ART was associated with having attended an antenatal clinic somewhere other than the study site (aOR: 4.7; 95% CI: 1.5–14.8) and with having been diagnosed with HIV during, rather than before, the index pregnancy (aOR: 3.2; 95% CI: 1.3–8.0).

**Table 3:** Factors associated with mothers known to be infected with HIV missing their uptake of antiretroviral therapy or missing nevirapine prophylaxis for their infants, Malawi, October 2014–May 2016

Characteristic	Unweighted denominator	Missed maternal ART uptake^a^			Missed infant nevirapine prophylaxis^a^	
		Unweighted, no. (%)	Weighted aOR^b^ (95% CI)		Unweighted, no. (%)	Weighted aOR^b^ (95% CI)
**Location (*n* = 3 233)**						
Rural facility in Central or North regions	803	9 (1.1)	1.5 (0.6–4.0)		63 (7.8)	1.5 (0.8–3.0)
Urban facility in Central or North regions	857	19 (2.2)	0.8 (0.3–2.3)		69 (8.1)	1.1 (0.5–2.4)
Rural facility in South region	881	9 (1.0)	1.3 (0.4–4.4)		59 (6.7)	1.3 (0.6–2.5)
Urban facility in South region	692	13 (1.9)	Reference		43 (6.2)	Reference
**Mother’s age in years (*n* = 3 224)**						
≤ 19	191	3 (1.6)	1.5 (0.1–14.1)		20 (10.5)	1.9 (0.7–4.8)
20–24	617	16 (2.6)	1.2 (0.2–5.6)		60 (9.7)	3.1 (1.8–5.5)
25–29	790	14 (1.8)	0.6 (0.1–3.3)		52 (6.6)	1.8 (1.4–2.4)
≥ 30	1626	17 (1.0)	Reference		100 (6.2)	Reference
**Parity (*n* = 3 230)**						
1	401	8 (2.0)	1.8 (0.4–8.5)		40 (10.0)	0.5 (0.2–1.2)
2–3	1328	27 (2.0)	1.1(0.3–3.2)		95 (7.2)	0.6 (0.2–1.5)
≥ 4	1501	15 (1.0)	Reference		98 (6.5)	Reference
**ANC uptake (*n* = 3 233)**			NIIM^c^			NIIM
Yes	3225	50 (1.6)			233 (7.2)	
No	8	0 (0.0)			1 (12.5)	
**Location of ANC (*n* = 3 225)**						
At study site	2559	24 (0.9)	Reference		172 (6.7)	Reference
At different site	666	26 (3.9)	4.7 (1.5–14.8)		61 (9.2)	1.9 (1.1–3.3)
**HIV status ascertained at ANC**^a^ **(*n* = 3 233)**						
Known HIV-positive before index pregnancy	1637	9 (0.5)	Reference		100 (6.1)	Reference
Found HIV-positive during index pregnancy	1596	41 (2.6)	3.2 (1.3–8.0)		134 (8.4)	0.8 (0.5–1.6)
**ART status and timing of ART initiation among confirmed HIV-infected mothers (*n* = 3 180)**						
On ART since before index pregnancy	1572	N/A	N/A		92 (5.9)	Reference
On ART, having started during index pregnancy	1475	N/A	N/A		76 (5.2)	0.6 (0.3–1.3)
On ART, having started postpartum	49	N/A	N/A		5 (10.2)	1.4 (0.3–6.5)
Off ART, having started and stopped	34	N/A	N/A		7 (20.6)	6.4 (1.5–28.0)
Off ART and never started	50	50 (100)	N/A		23 (46.0)	15.4 (7.2–32.9)

ANC: antenatal clinic; aOR: adjusted odds ratio; ART: antiretroviral therapy; CI: confidence interval; HIV: human immunodeficiency virus; N/A: not applicable; NIIM: not included in model.

^a^ As reported by mothers 4–26 weeks postpartum.

^b^ Weighted according to sampling interval and the probabilities of districts, clusters and subjects being selected and adjusted for all other variables in the model.

^c^ Too few (0) events to be included in the model.

Among all known HIV-exposed infants, 234 (7.7%) had reportedly not received any nevirapine prophylaxis (Table 3). An infant that had missed prophylaxis was more likely to have: (i) a mother aged 20–24 (aOR: 3.1; 95% CI: 1.8–5.5) or 25–29 (aOR: 1.8; 95% CI: 1.4–2.4) years than one older than 30 years; (ii) a mother who had attended an antenatal clinic somewhere other than the study site than a mother who had received antenatal care at the study site (aOR: 1.9; 95% CI: 1.1–3.3); and (iii) a mother who had either stopped ART (aOR: 6.4; 95% CI: 1.5–28.0) or never started ART (aOR: 15.4; 95% CI: 7.2–32.9) than a mother on ART.

Overall, 675 (46.8%) of all known HIV-exposed infants that were older than eight weeks when their mothers were interviewed had reportedly not been tested for HIV-1 DNA at that time (Table 4). Mothers with infants that had not been tested were more likely to be younger than 19 years (aOR: 6.9; 95% CI: 1.2–38.9) or aged 20–24 (aOR: 2.7; 95% CI: 1.3–5.6) or 25–29 years (aOR: 1.8; 95% CI: 1.1–3.0) than to be aged at least 30 years. They were also more likely to have a parity of at least 4 (aOR: 3.3; 95% CI: 1.1–10.1) than be primiparous, to have stopped (aOR: 7.7; 95% CI: 2.2–27.0) or never started ART (aOR: 13.7; 95% CI: 4.2–83.3) than to be on ART and to have given no nevirapine prophylaxis to their infants than to have given such prophylaxis (aOR: 2.0; 95% CI: 1.2–3.4; Table 4).

**Table 4:** Factors associated with missed infant virological testing among infants over 8 weeks of age who had been born to mothers known to be HIV infected, Malawi, October 2014–May 2016

Characteristic	Unweighted denominator	Missed infant testing^a^	
		Unweighted, no. (%)	Weighted aOR^b^ (95% CI)
**Location (*n* = 1 465)**			
Rural facility in Central or North regions	344	166 (48.3)	1.3 (0.5–3.3)
Urban facility in Central or North regions	402	146 (36.3)	0.8 (0.5–1.5)
Rural facility in South region	444	251 (56.5)	1.6 (0.9–3.0)
Urban facility in South region	275	112 (40.7)	Reference
**Mother’s age in years (*n* = 1 462)**			
≤ 19	87	48 (55.2)	6.9 (1.2–38.9)
20–24	272	130 (47.8)	2.7 (1.3–5.6)
25–29	366	174 (47.5)	1.8 (1.1–3.0)
≥ 30	737	322 (43.7)	Reference
**Parity (*n* = 1 464)**			
1	179	82 (45.8)	Reference
2–3	586	253 (43.2)	1.4 (0.6–3.3)
≥ 4	699	340 (48.6)	3.3 (1.1–10.1)
**ANC uptake (*n* = 1 465)**			NIIM
Yes	1460	674 (46.2)	
No	5	1 (20.0)	
**ANC location (*n* = 1 459)**			
At study site	1109	505 (45.5)	Reference
At different site	350	168 (48.0)	0.9 (0.6–5.6)
**HIV status ascertained at ANC (*n* = 1 465)**			
Known HIV-positive before index pregnancy	724	312 (43.1)	Reference
Found HIV-positive during index pregnancy	741	363 (49.0)	1.8 (0.6–1.4)
**ART status and timing of ART initiation among confirmed HIV-infected mothers (*n* = 1 435)**			
On ART since before index pregnancy	692	297 (42.9)	Reference
On ART, having started during index pregnancy	672	303 (45.1)	0.7 (0.2–2.1)
On ART, having started postpartum	23	8 (34.8)	0.2 (0.1–0.8)
Off ART, having started and stopped	21	18 (85.7)	7.7 (2.2–27.0)
Off ART and never started	27	23 (85.2)	13.7 (4.2–83.3)
**Infant nevirapine prophylaxis given to known HIV-exposed infants (*n* = 1 465)**			
From birth to an age of 6 weeks	1241	522 (42.1)	Reference
For less than 6 weeks	95	49 (51.6)	0.8 (0.4–1.4)
Not given or unknown	129	104 (80.6)	2.0 (1.2–3.4)

ANC: antenatal clinic; aOR: adjusted odds ratio; ART: antiretroviral therapy; CI: confidence interval; HIV: human immunodeficiency virus; NIIM: not included in model.

^a^ As reported by mothers at least 8 weeks postpartum.

^b^ Weighted according to sampling interval and the probabilities of districts, clusters and subjects being selected and adjusted for all other variables in the model.

### Programme use and MTCT

**Fig. 1:**
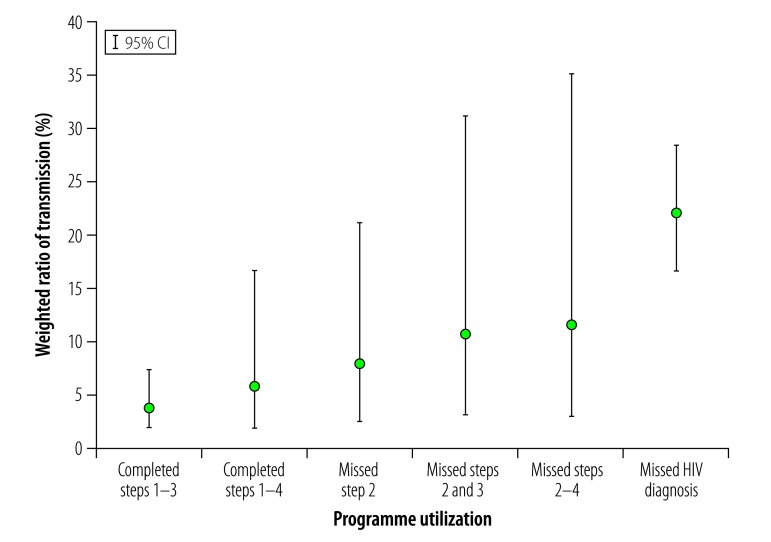
Ratio of mother-to-child transmission of HIV, as recorded 4–26 weeks postpartum, Malawi, October 2014–May 2016

The relationship between MTCT ratios and the missing of one or more steps in the cascade of care is illustrated in Fig. 1. Among the mothers who missed HIV diagnosis, the MTCT ratio was 22.0% (66/286; 95% CI: 16.6–28.4). Of the 3233 confirmed HIV-infected mothers, 2613 (74.7%; 95% CI: 64.6–82.7) completed steps 1–3. The MTCT ratio for these 2613 mothers was 3.6% (66/2613; 95% CI: 1.8–7.3). Among the 1435 mothers who, when interviewed, had known HIV-exposed infants older than eight weeks, 711 (39.2%; 95% CI: 32.4–46.4) completed steps 1–4. The MTCT ratio for these 711 mothers was 5.7% (19/711; 95% CI: 1.8–16.7).

CI: confidence interval.

Notes: Ratios were recorded for the mother–infant pairs who missed or did not miss some of the main steps along the cascade of care provided by the national programme for the prevention of mother-to-child transmission of human immunodeficiency virus. Step 1: ascertainment of HIV status during antenatal care; step 2: uptake of maternal ART; step 3: use of infant nevirapine prophylaxis: step 4: HIV testing, before the study, of HIV-exposed infants when more than eight weeks old. The values were weighted according to sampling interval and the probabilities of districts, clusters and subjects being selected.

Among the 50 mothers who missed step 2, MTCT was 7.7% (6/50; 95% CI: 2.5–21.2). Steps 2 and 3 were both missed by 23 (0.7%; 95% CI: 0.3–1.6) mothers and their MTCT ratio was 10.7% (5/23; 95% CI: 3.1–31.3). Just 14 (1.2%; 95% CI: 0.5–3.1) of the 1435 mothers who, when interviewed, had known HIV-exposed infants aged over eight weeks had reportedly missed steps 2–4 and their MTCT ratio was 11.4% (4/14; 95% CI: 3.0–35.2).

The MTCT ratio among mothers who had missed HIV diagnosis was significantly higher than that among mothers who had completed steps 1–3 (*P* < 0.01) or 1–4 (*P* < 0.01) or missed step 2 (*P* = 0.04). None of the other differences seen between MTCT ratios, e.g. between mothers who had completed and missed steps or between mothers who had missed HIV diagnosis and those who had missed steps 2–3 or 2–4, reached statistical significance.

## Discussion

We estimated that 12.1% of infants of women attending under-5 clinics across Malawi were HIV-exposed. This value is higher than an aggregated estimate (8.1%) based on data collected in antenatal-care facilities across Malawi,^8^ but consistent with the prevalence of HIV infection reported among women aged 15–49 years (12.1%) as part of the recent HIV impact assessment in Malawi.^14^

The high uptake of PMTCT services we report aligns with estimates produced by the health minstry.^14^^,^^15^ The PMTCT uptake was similar across regions or between rural and urban sites. Our results add to the evidence indicating that, within Malawi, the scale-up of the implementation of the Option B+ strategy has provided widely decentralized and equitable coverage of PMTCT services.^8^^,^^16^

The elimination of paediatric HIV infections will probably depend on the full use of PMTCT services.^17^^–^^19^ In South Africa, a third of early infant HIV infections were attributed to the missing of one or more steps in the PMTCT cascade of care and, as observed in our study, the MTCT ratio increased as more steps were missed.^10^

We found that young women and adolescent girls were particularly prone to missing antenatal HIV testing. Data previously collected in Kenya,^20^ Malawi,^21^ and South Africa^10^^,^^22^ indicated that adolescents were especially likely to miss parts of the PMTCT cascade.^10^^,^^20^^–^^22^ In our study, adolescents found HIV-positive usually started ART and infant nevirapine prophylaxis, but often missed infant HIV testing. Further research is needed to improve our understanding of the retention of, and outcomes among, young women and to assess the potential benefits of youth-friendly initiatives such as peer-support groups for pregnant adolescents.

We identified a substantial number of women who missed HIV diagnosis during the index pregnancy and these were associated with a relatively high MTCT ratio. Some of these women may have had the very high viral loads during acute HIV infection that are strongly associated with MTCT.^23^ We need more research on the identification and engagement of this high-risk subgroup.

The impact of service integration and timing of HIV diagnosis on uptake of ART was demonstrated by the fact that HIV-infected women were relatively unlikely to be on ART if they had received antenatal care at a different facility to the one hosting the under-5 clinic they attended and if they had been found HIV-infected during, rather than before, the index pregnancy. In earlier studies in Malawi, a facility’s model of care was found to influence mothers’ engagement in the PMTCT cascade^24^ and retention on ART has been found to be relatively poor in facilities where women were asked to initiate ART on the same day that they discovered they were HIV-infected.^25^ For the women involved, HIV diagnosis during pregnancy and the concept of life-long treatment are both challenging.^26^^,^^27^ Further understanding of the impact of a HIV diagnosis during pregnancy and of the optimal model of care is required.

In our study, around a quarter of HIV-exposed infants did not receive the full six-week course of nevirapine prophylaxis and almost half of the HIV-exposed infants above eight weeks of age had not been tested for HIV-1 DNA. Delayed or missed infant HIV diagnoses, which have previously been highlighted as key challenges in Malawi,^8^^,^^28^ lead to delayed ART and place HIV-infected infants at high risk.

If we project our findings to the Malawian population and burden of HIV, we estimate that, in 2017, about 16 874 Malawian women, i.e. 2.2% of the 767 000 Malawian women who gave birth,^29^ did not receive antenatal HIV testing. If we assume a 12.1% prevalence of HIV infection among the fertile women,^14^ we can estimate that 92 807 pregnant Malawian women were HIV-infected, including 6589 (7.1%) who missed HIV diagnosis in pregnancy. Among the women who knew that they were HIV-positive when pregnant, an estimated 1035 (1.2%) will not have started ART, 6639 (7.7%) of their infants will not have received any nevirapine prophylaxis and 40 350 (46.8%) of their infants will not have been tested for HIV-1 DNA after they were aged six weeks. An estimated 69 327 (74.7%) of the mothers will have received antenatal HIV testing and, if found infected, started maternal ART and given nevirapine prophylaxis to their infants, of whom an estimated 2496 (3.6%) will have been infected. Among the 6589 mothers who missed HIV diagnosis, 1450 (22.0%) will have transmitted HIV to their infants. Based on our findings and these projections, the focus of future targeted interventions needs to be on reducing the numbers of missed HIV diagnoses in pregnancy and increasing the proportion of HIV-exposed infants tested for HIV-1 DNA.

The large sample size and the study’s national representativeness are strengths of this study. However, by screening at under-5 clinics, we may have biased our sample towards women who usually take up health care. We made no attempt to recruit women who do not attend clinics and those with infants who died younger than four weeks. As a result, our estimates of the use of the PMTCT programme may be too high. We also acknowledge the potential for responder bias in terms of reported uptake of services, although interviewers checked mothers’ health booklets to confirm responses. Lastly, as some of the numbers in the subgroup analyses were small, the reported confidence intervals are wide and need to be interpreted with care. The same small numbers limited the statistical significance of the between-subgroup differences seen in MTCT ratios.

In conclusion, the scale-up of Option B+ services appears to have provided universal access to PMTCT care. The findings that young maternal age, high parity and use of services at different clinics were associated with incomplete care should help to guide the development of interventions to accelerate progress towards the elimination of MTCT from Malawi.
